# Identification of Maturity-Onset-Diabetes of the Young (MODY) mutations in a country where diabetes is endemic

**DOI:** 10.1038/s41598-021-95552-z

**Published:** 2021-08-09

**Authors:** Hessa Al-Kandari, Dalia Al-Abdulrazzaq, Lena Davidsson, Rasheeba Nizam, Sindhu Jacob, Motasem Melhem, Sumi Elsa John, Fahd Al-Mulla

**Affiliations:** 1 Department of Population Health, Dasman Diabetes Institute, Kuwait City, Kuwait; 2 Department of Pediatrics, Farwaniya Hospital, Ministry of Health, Kuwait City, Kuwait; 3 Department of Pediatrics, Faculty of Medicine, Kuwait University, Kuwait City, Kuwait; 4 Department of Genetics and Bioinformatics, Dasman Diabetes Institute, Kuwait City, Kuwait

**Keywords:** Clinical genetics, Disease genetics, Genetic testing

## Abstract

Genetic variants responsible for Maturity-Onset-Diabetes of the Young (MODY) in Kuwait were investigated. A newly established a National Referral Clinic, the Dasman Diabetes Institute (DDI-NRC), assessed forty-five members from 31 suspected MODY families by whole exome sequencing. Thirty-three of the 45 samples were independently sequenced at the DDI-NRI, Exeter University, UK (https://www.diabetesgenes.org/) using targeted 21-gene panel approach. Pathogenic mutations in GCK, HNF1A, HNF1B, HNF4A, and PDX1 confirmed MODY in 7 families, giving an overall positivity rate of 22.6% in this cohort. Novel variants were identified in three families in PDX1, HNF1B, and HNF1B. In this cohort, Multiplex Ligation-dependent Probe Amplification assay did not add any value to MODY variant detection rate in sequencing negative cases. In highly selected familial autoantibody negative diabetes, known MODY genes represent a minority and 77.3% of the familial cases have yet to have a causal variant described.

## Introduction

Maturity-onset diabetes of the young (MODY) is a rare form of monogenic diabetes caused by dominantly acting heterozygous variants in genes necessary for the development or function of pancreatic β-cells^1^. To date, multiple distinct forms of MODY (MODY1-14) have been identified with varied clinical and genetic heterogeneity^2^. MODY frequently represents a diagnostic challenge for clinicians as it is a rare condition that shares clinical features with both type 1^3,4^, and type 2 diabetes mellitus^5,6^ (T1D and T2D) and is often misdiagnosed as such. While clinicians often learn about MODY from its autosomal dominant inheritance pattern, many individuals lack a family history^7^. However, unlike T1D and T2D, molecular genetic testing is sensitive and specific for diagnosing MODY, and it provides essential information on therapeutic management and follow-up^8–10^.

The 14 known MODY genes account for ~ 1–6% of pediatric diabetes cases worldwide^11,12^. Since dozens of disease alleles have been reported for most of these loci in European and non-European populations^13^, next generation sequencing (NGS) based genetic testing is the preferred diagnostic approach as it provides cost-effective screening of the entire exome or a selected panel of MODY genes^14^. The genetic changes associated with MODY in European populations have expanded to include copy number variation^15^. As a result, the introduction of additional technologies, such as DNA microarrays or Multiplex Ligation-dependent Probe Amplification (MLPA), can complement NGS in establishing a definitive diagnosis of MODY.

To date, most studies have searched for genetic causes of MODY in individuals with European ancestry, while only a small number of studies have been conducted in Arabia and the Middle East^16–20^. Given the high frequency of consanguineous marriages and the large burden of genetic homozygosity^21^, studies in this region are crucial to identify novel genetic variants and new MODY-associated genes. Recently, we highlighted the MODY knowledge gap to healthcare providers in the Middle East region to rectify this niche^16^. To the best of our knowledge, this is the first study from the region reporting on an initiative to diagnose patients with MODY based on a referral system to a specialized National Referral Clinic at the Dasman Diabetes Institute (DDI-NRC). We present data from the initial phase of the study, which describes 31 families characterized by whole exome sequencing (WES) and MLPA performed at the DDI-NRC and by a model sequencing panel performed independently at the Royal Devon and Exeter NHS Foundation Trust Genetics Laboratory (“Exeter”).

## Materials and methods

### Family data and diagnosis

This study reports data collected between January 2013 to June 2017. Patients with suspected MODY (31 families, 45 individuals), based on the International Society of Pediatric and Adolescent Diabetes (ISPAD) clinical criteria^9^, were referred from all over Kuwait to the DDI-NRC. At the DDI-NRC, the patients and their family members completed a standardized questionnaire and were evaluated by a pediatric endocrinologist, who examined the patients, collected detailed information on their phenotype, and coordinated with the treating physicians to compile clinical data. Body mass index (BMI) measures for children younger than the age of 19 years were expressed as standard deviation scores (SDS) determined by the World Health Organization growth standards^22^.

MODY was suspected if: (1) the patient was < 25 years of age at the time of diabetes diagnosis; (2) had a family history suggestive of autosomal dominant inheritance; (3) lacked evidence suggesting a diagnosis of T1D (evidence of endogenous insulin production outside the honeymoon period with detectable C peptide level (> 200 nmol/L) when glucose is > 8 mmol/L and absence of pancreatic islet autoantibodies); and/or (4) lacked evidence supporting a diagnosis of T2D (normal body weight, absence of acanthosis nigricans, and no evidence of insulin resistance with normal fasting C peptide level).

The index patient and/or their parents were informed about the benefits of testing first-degree relatives: if consent was provided, family members were also enrolled. Genetic testing was performed in Kuwait (DDI) and at Exeter. The DDI laboratory and bioinformatic staff were blinded to the Exeter results. Only the Principal Investigator of the study had full access to the laboratory results. Patients with positive test results were offered genetic counselling and susceptibility testing of first-degree relatives, if not already performed.

The study was approved by the Ethical Review Committees at DDI and the Ministry of Health in Kuwait and carried out in accordance with the principles of the Declaration of Helsinki as revised in 2008. Written informed consent was obtained from all patients or their parents if the index patient was a minor.

### Extraction of DNA from peripheral blood

Blood samples were collected from the index patient and his/her first-degree relatives. Genomic DNA was extracted using a QIAamp Blood DNA kit (Qiagen, Germany) and quantified spectrophotometrically using a Qubit Fluorometer (Thermofisher, USA), following the manufacturer’s protocol.

### Whole exome sequencing and analysis

Exome libraries were prepared using Nextera Rapid Capture Exome kits (Illumina Inc., USA) following the manufacturer’s protocol. Sequencing was carried out on a HiSeq 2500 using Illumina’s Sequence by Synthesis technology with 100 bp paired-end reads at a mean depth of 100X.—BCL to FASTQ conversion was carried out using bcl2fastq v.2.20 software. Sequence reads are aligned to the reference human genome build hg19 using BWA v.0.7.17, and variant calling was carried using GATK v.3.7 HaplotypeCaller following the best practices. The variant call file of the affected individual was analyzed using VSClinical v2.2.2 software (Golden Helix, MT, USA) with default setting of genotype quality of ≥ 20, depth ≥ 25, and MAF OF < 0.01. Of the variants that pass the filter, only those that are predicted to cause loss-of-function or missense mutations were analyzed. The pathogenicity of identified variants was further assessed using ACMG criteria. Allelic frequencies were estimated, within Varseq, based on public databases including gnomAD v2.1.1, dbSNP, 1000 Genomes Project, our local Arab genome database, and Iranome^23^. Clinical interpretation of the variants was based on Polyphen, ClinVar, Leiden Open Variation Database (LOVD), Human Gene Mutation Database (HGMD), Online Mendelian Inheritance in Man (OMIM), and American College of Medical Genetics (ACMG) recommendations. The quality of the sequence reads was assessed using Integrative Genomics Viewer (IGV) Software (Broad Institute, United States).

### Targeted validation by sanger sequencing

Shortlisted variants were validated by Sanger sequencing on an ABI 3730 xl DNA sequencer (Applied Biosystems, Foster City, USA) following standard protocol.

### Multiplex Ligation-dependent Probe Amplification (MLPA) assay

MLPA assays were carried out following the manufacturer’s protocol (MRC-Holland, Amsterdam, The Netherlands) using P241-E1 MODY Mix-1 and P357-A3 MODY Mix-2. A total of 100 ng of DNA extracted from peripheral blood of patients, and reference samples were subjected to denaturation, hybridization, ligation, and amplification as per protocol instructions. Capillary electrophoresis was performed on ABI-3730xl DNA Analyser (Applied Biosystems, USA), and data were analyzed using Coffalyser (MRC Holland).

## Results

### Defining the MODY phenotypic spectrum

During the study period, 31 families (45 individuals) were recruited and referred for genetic screening. Table 1 shows the clinical characteristics of the index patients (16 males and 15 females); mean age was 13.6 (SD ± 7.48) years, mean age at diagnosis of diabetes was 10.02 (SD ± 5.85) years, and mean BMI z scores was 1.37 (SD ± 1.42). The mean HbA1c for the index patients was 8.26% (SD ± 1.73).

**Table 1:** Clinical and genetic characterization of index cases of MODY families.

Family No	Total No. of family members	No. of family members suspected with MODY	Age of index case (years)	Age at Diagnosis (years)		Gender (M/F)	BMI (kg/m^2^) Z score (SD)	HbA1C (%)
1	12	5	18	16		M	25.7 (1.55)	9.3
2	22	5	11.6	7		M	19.3 (2.13)	8.5
3	42	10	30	16		F	23.2 (0.75)	7.4
4	16	4	10.4	8		F	15.0 (− 0.38)	11.7
5	10	3	12.2	11		M	15.3 (− 1)	6.3
6	8	1	13.7	11		M	22.5 (2)	7.6
7	2	2	15.9	13.9		M	16.9 (0.99)	7.3
8	20	10	6	2.5		F	18.1 (1.72)	7.4
9	15	10	26.3	22		F	25.5 (NA)	–
10	14	7	17	15		F	26.6 (1.71)	5.2
11	14	6	12	7		F	18.7 (1.61)	11.9
12	10	8	6.4	5.8		M	15.1 (− 0.1)	6.9
13	12	4	8.3	6		M	17.9 (1.68)	7.3
14	13	7	13.2	9		M	20.2 (1.9)	–
15	22	12	8.9	8		M	19.3 (1.85)	7.7
16	20	8	8.7	7		F	13.9 (− 0.99)	–
17	12	4	6.2	0.11		M	17.8 (1.79)	8.5
18	17	12	40.2	28		F	22.1 (NA)	10.1
19	14	7	16.6	16.3		M	22.4 (0.62)	9.9
20	15	10	8.1	3.5		F	15.4 (0.04)	8
21	22	11	14.6	12		F	31.5 (2.95)	6.5
22	14	5	6.8	6		M	14.3 (− 0.83)	8.2
23	33	17	14.8	11		F	25.7 (2.38)	12.7
24	19	11	12	11		M	24.1 (2.37)	7.1
25	14	7	9.9	9		F	18.9 (1.2)	7.1
26	21	7	10.8	10		F	24.5 (2.41)	7.2
27	10	4	6.7	3.5		M	23.9 (5.46)	8.4
28	15	10	12.4	12		M	26.1 (2.47)	–
29	15	7	12.9	12		F	21.3 (1.15)	6.4
30	30	9	10	9.1		M	24.4 (3)	9.5
31	32	18	22.1	2		F	17.9 (1.47)	8.5

*BMI* Body mass index, *HbA1C* Hemoglobin A1C.

### The MODY exome and validation

Forty-five patients contributed DNA for exome sequencing (31 index patients and 15 first-degree family members). Seven out of 31 index patients (22.6%) were confirmed as MODY with variants in genes encoding *GCK, HNF1A, HNF1B, HNF4A*, and *PDX1* (Table 2). In most cases (24 families; 77%), no variant of significance was detected in any monogenic diabetes-linked genes (Table 2). To exclude large deletion/duplication events as a possible cause of MODY in these 24 families, we subjected the DNA to MLPA but found no copy number variant in any of the MODY genes (Fig. S1). To confirm our sequencing and MLPA data accuracy, 35 samples from 28 families were independently validated by comparing the DDI-NRC results with panel-based genetic testing performed at Exeter (adopting a blinded experimental approach).

**Table 2:** Genetic variants identified in seven families with MODY.

Family No	Gene/MODY	Chromosome/location	DNA change	Protein change	MAF	SNP ID	SIFT/Polyphen Prediction	Z-score	Genetic mutation in family members	Family history of DM	Treatment	ACMG classification	ACMG **prediction**
1	PDX1 MODY 4	Chr13/	Heterozygous	NP_000200.1:	NR	Novel	Damaging	3.13	Confirmed in index case & his affected brother	Yes	Insulin therapy	PM2, PP1, PP2,	Likely pathogenic
			NM_000209.3:									PP3, PP4	
												1 Moderate AND	
		28498447	c.461C > G	p. Thr154Arg								≥ 4 supporting	
2	HNF1B MODY 5	Chr17/	Heterozygous	NP_000449.1:	NR	Novel	Damaging	1.73	Confirmed in index case		Insulin therapy	PM1, PM2, PP2, PP3, PP4	Likely pathogenic
			NM_000458.3:									2 Moderate AND	
		36099563	c.412G > A	p. Glu138Lys								≥ 2 supporting	
27	HNF1B MODY 5	Chr17/	Heterozygous	NP_000449.1:	NR	rs121918675	Damaging	2.32	Confirmed in index case		Insulin therapy	PS1, PM1, PM2, PM5, PP2, PP3, 1 Strong, 3 Moderate	Pathogenic
		36099481	NM_000458.4:	p. Arg165His									
			c.494G > A										
3	HNF1A MODY3	Chr12/	Heterozygous		0.00001	rs587776825	–		Confirmed in index case		Insulin therapy replaced by Oral agent	PVS1, PM4, PP3, PP4, PP5 1 Very strong (PVS1) AND 1 Moderate and 1 supporting	Pathogenic
			NM_000545.6:	Gly292Argfs*25									
		121432116	c.872dupC	Loss of function									
9	HNF1A MODY3	Chr12/	Heterozygous	NP_000536.5:	NR	Novel	Damaging	1.14	Confirmed in index case, her affected mother & 3 siblings	Yes	Oral agent	PM2, PP1, PP2, PP3, PP4 1 Moderate AND ≥ 4 supporting	Likely pathogenic
			NM_000545.6:										
		121416579	c.8C > A	p. Ser3Tyr									
8	HNF4A MODY1	Chr20/	Heterozygous		0.00037	rs147638455	Tolerated by Polyphen	1.81	Confirmed in index case	Maternal history of GDM		BS1, PP2 Criteria not met	VUS
			NM_000457.4:	NP_000448.3:									
		43058267	c.1387A > G	p. Ile463Val									
12	GCK MODY2	Chr7/	Heterozygous		NR	rs886042610	Damaging	3.07	Confirmed in index case & his affected mother	Yes		PM1, PM2, PM5, PP1, PP2, PP3, PP4 ≥ 3 Moderate	Likely pathogenic
			NM_000162.5:	NP_000153.1:									
		44189575	c.572G > A	p. Arg191Gln									

MAF, Minor allele frequency; VUS, Variant of unknown significance, *Z-score indicates a gene's intolerance to missense variants. Z-score >1 indicates a low rate of benign missense change of the gene or increased intolerance/constraint to variation. NR denotes Not Reported in Public database. ACMG classification was carried out following the recommendations of Sue Richards et al. (2015).

PVS1, Very strong evidence of pathogenicity.

PS1-PS2-PS3-PS4, Strong evidence of pathogenicity.

PM1-PM2-PM3-PM4-PM5, Moderate evidence of pathogenicity.

PP1-PP2-PP3-PP4-PP5, Supporting evidence of pathogenicity.

BA1, stand-alone evidence of benign impact.

BS1-BS2-BS3-BS4, Strong evidence of benign impact.

BP1-BP2-BP3-BP4-BP5-BP6-BP7, Supporting evidence of benign impact.

### Identification of causal variants in MODY

A likely causal variant was identified in 21.8% of the studied families, spanning 36 genes that have been reported previously to cause monogenic diabetes. Each of the seven affected individuals carried a monoallelic variant in one of the 36 monogenic diabetes genes, and together represented five different subtypes of MODY (Table 2).

**Figure 1:**
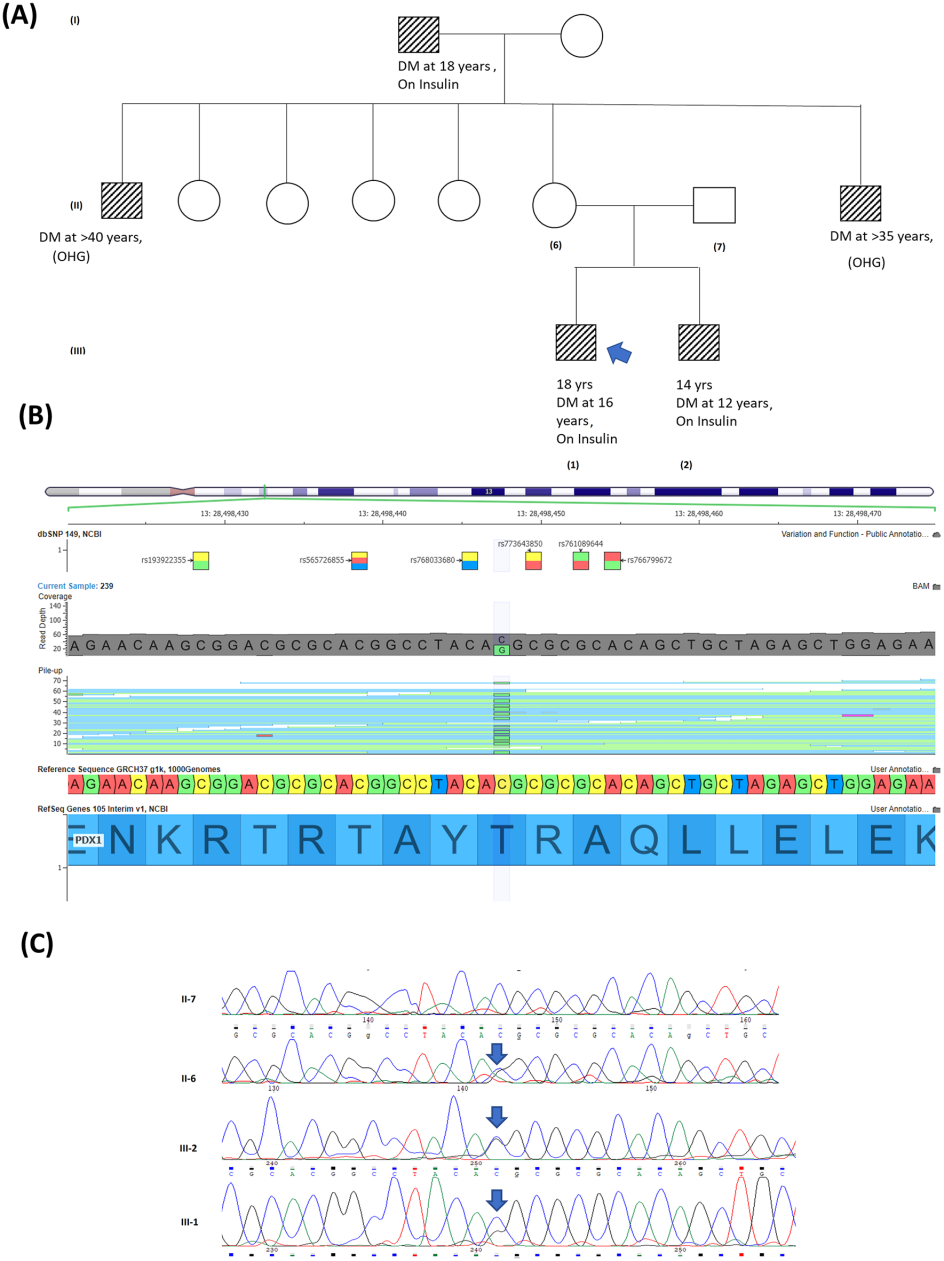
(**A**) Represents pedigree of a family positive for Maturity Onset Diabetes of the Young (MODY4). The square symbol represents male, and the circle represents female. The filled in symbols indicate affected individuals and the blue arrow indicates the index case. Next generation sequencing revealed a novel heterozygous missense variant in Pancreatic and duodenal homeobox 1 (PDX1) c.461C > G resulting in p.Thr154Arg. (**B**) Exome sequencing summary layout depicting part of the PDX1 gene location on chromosome 13 at the top and the exons coverage in green and blue. The location of the variants in exon 2 is represented by a vertical green bar and the reference sequence and the altered codon is highlighted. (C) Chromatogram represents Sanger sequencing results confirming the segregation of heterozygous variants in the mother, the index case and his brother.

In Family 1, two male siblings presented with diabetes at the ages of 16 and 12 years, respectively, requiring insulin replacement therapy (Fig. 1A). Exome sequences from both siblings showed a novel missense variant in *PDX1*, which was confirmed by Sanger sequencing in both siblings (Fig. 1B,C). The maternal grandfather, as well as two maternal uncles, had diabetes, indicating a likely possibility of maternal transmission of the variant. Indeed, the variant was maternally inherited, although the mother was not diabetic, suggesting incomplete penetrance of this variant (Fig. 1C). Incomplete penetrance of pathogenic mutations in the *PDX1* gene has been described previously^24^, particularly in the absence of *MLKL* genetic mutation, a key necroptosis protein^25^. *PDX1* is an essential gene in pancreatic development: homozygous *PDX1* variants have been previously described in two children with pancreatic agenesis (one who had consanguineous parents and one whose both parents had MODY4);. In contrast, heterozygous *PDX1* variants have been shown to cause MODY4 in a small number of studies^26^. Our finding thus reports a novel *PDX1* variant. Although we used the objective ACMG scoring system, which designated it as “likely pathogenic”, we need to be cautious with this label because the variant has not been observed previously, and functional studies are lacking. Clinvar reports six known pathogenic missense variants in *PDX1*, indicating that missense alterations are a common disease-causing mechanism in this gene. The c.461C > G, p.Thr154Arg variant is located in the DNA-binding homeodomain and is two amino acids downstream of T151, which is phosphorylated by Per-Arnt-Sim Kinase (PASK) and is essential for the regulation of insulin promoter factor-1 activity.

The rare clinical subtype, MODY5, was confirmed by identifying variants in *HNF1B* for two index cases whose clinical presentations were consistent with the diagnosis of renal cyst and diabetes syndrome (RCAD) (Table 2). The *HNF1B* exon 4 p.E138K variant, in family 2, was detected in a Kuwaiti boy diagnosed shortly after birth with chronic renal failure due to bilateral cystic kidney disease. After renal transplantation at the age of 3 years, he started to show intermittent symptomatic hyperglycemia at the age of 9 years. His hyperglycemia became persistent, requiring insulin therapy at the age of 10 years. Unfortunately, other family members refused to consent for genetic testing. Three variants within six amino acid positions of the variant p.E138K have been shown to be pathogenic, while none are benign. The second *HNF1B* variant, c.494G > A, was identified in 6 years and nine-month-old Pakistani boy who was found to have a high creatinine level (108.0 µmol/L) at the initial diagnosis of diabetes at the age of around 4- years. The child was obese with a BMI of 23.9 kg/m^2^ (z score + 3.84 SD) and was managed by multiple daily injections of insulin (Table 2, Family 27). The c.494G > A (p.R165H) variant has been reported as “pathogenic” and “likely pathogenic” in ClinVar (https://www.ncbi.nlm.nih.gov/clinvar/variation/12647/). Nine variants within six amino acids of p.R165H have been shown to be pathogenic, while none are benign. *HNF1B* contains 74 pathogenic missense variants, indicating that missense variants are a common disease mechanism in this gene. It encodes a member of the homeodomain-containing superfamily of transcription factors. The protein binds to DNA as either a homodimer or a heterodimer with the related protein hepatocyte nuclear factor 1-alpha (*HNF1A*). *HNF1B* has been shown to function in nephron development and to regulate the development of the embryonic pancreas. Variants in this gene result in renal cysts and MODY5.

**Figure 2:**
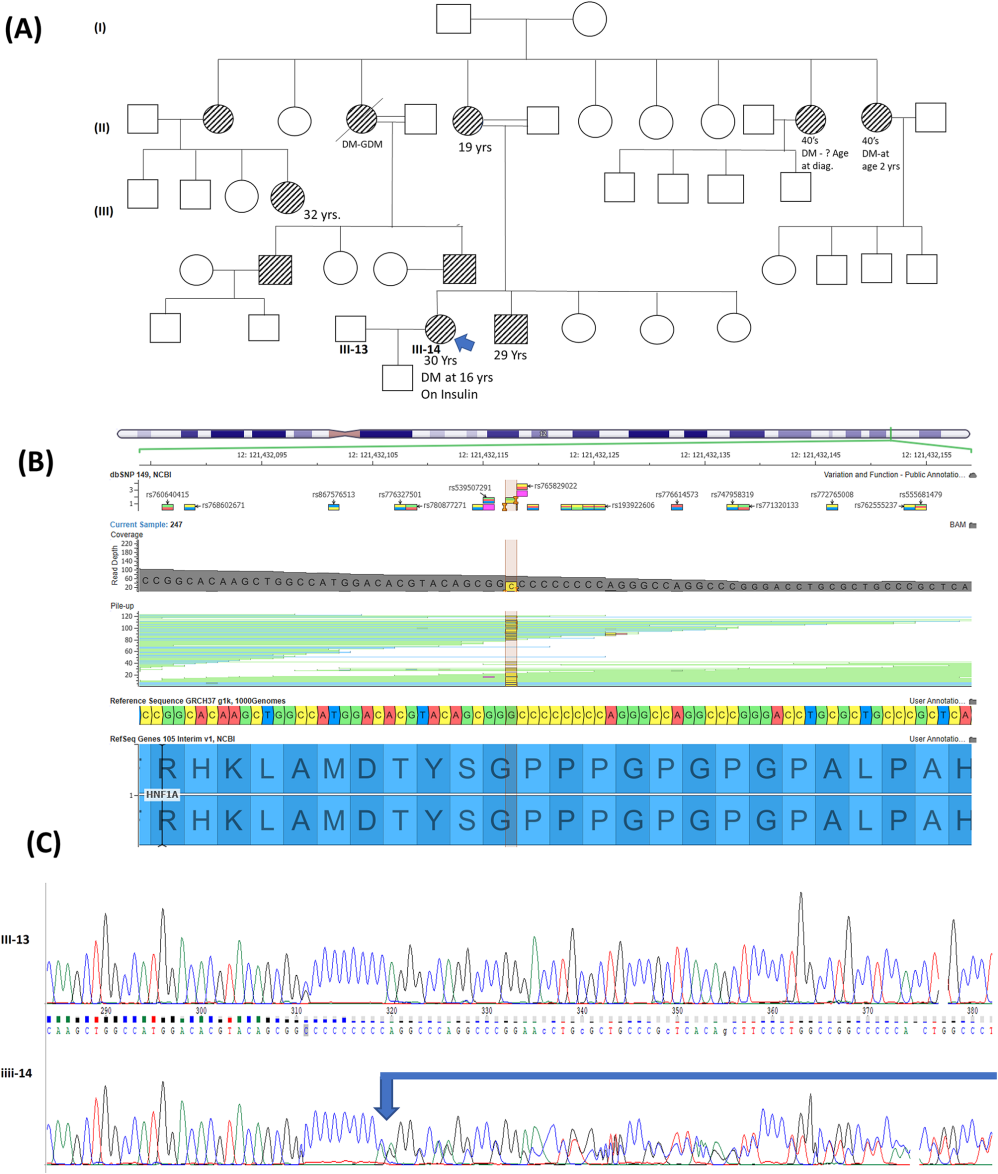
(**A**) Represents pedigree of a family positive for Maturity Onset Diabetes of the Young (MODY3). The square symbol represents male, and the circle represents female. The filled in symbols indicate affected individuals and the blue arrow indicates the index case. (**B**) Exome sequencing summary layout depicting part of the HNF1A gene location on chromosome 12 at the top and the exons coverage in green and blue. The location of the c.872dupC, (p.Gly292fs) frameshift mutation is represented by a vertical yellow bar and the reference sequence and the altered codon is highlighted. (**C**) Chromatogram represents Sanger sequencing of the HNF1A gene in control family member (top) and confirming the heterozygous frameshift mutation in the index (bottom arrow-line).

**Figure 3:**
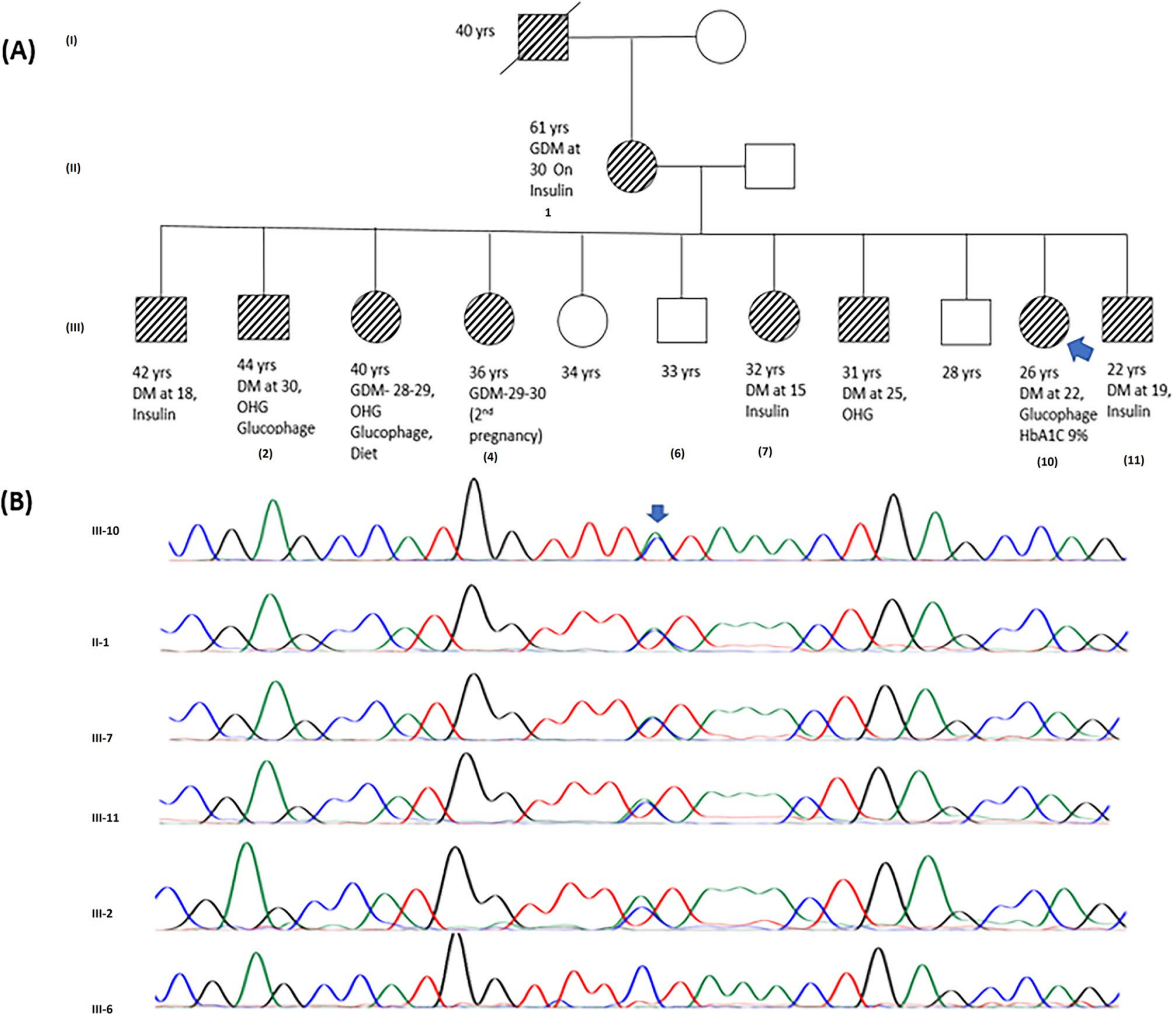
(**A**) Represents pedigree of a family positive for Maturity Onset Diabetes of the Young (MODY3). The square symbol represents male, and the circle represents female. The filled in symbols indicate affected individuals and the blue arrow indicates the index case. Next generation sequencing revealed a novel heterozygous missense variant in HNF1A, c.8C > A, resulting in p.Ser3Tyr. (**B**) Chromatogram represents Sanger sequencing results confirming the segregation of heterozygous variant in the index case, affected and unaffected family members.

The diagnosis of MODY3 (*HNF1A*) was established in two index cases, one with a well-known frameshift insertion c.872dupC (p. G292Argfs*25) in exon 4 and one with a novel heterozygous missense variant c.8C > A (p.S3Y) in exon 1 of the *HNF1A* (Table 2). The frameshift c.872dupC variant was detected in a 30-year-old Jordanian woman diagnosed with T1D at the age of 16 years (Fig. 2). She was initially treated with insulin (0.5 U/kg/day). Following the diagnosis of MODY3, the insulin therapy was replaced with Gliclazide (60 mg, twice daily), resulting in optimal glycemic control (HbA1c = 6.7%). This specific variant has been reported previously in at least 22 families and is listed in LOVD database 322 times as pathogenic. The variant causes a frameshift with loss of HNF1α protein activity. The pathogenic duplication variant detected in this family is estimated to account for approximately 20% of *HNF1A*-MODY3 families^27^. The other heterozygous c.8C > A variant in *HNF1A* was identified in a 26-year-old Kuwaiti woman diagnosed with T2D who had a strong family history of diabetes (Fig. 3). She was slightly overweight with a BMI of 25.5 kg/m^2^ and had poor glycemic control (HbA1c = 9.0%) while being treated with oral agents (Tables 1 and 2, Family 9). The missense variant p.S3Y in HNF1A has not been reported previously as a pathogenic variant nor as a benign variant, to our knowledge, but has been previously described in a patient with hepatocellular carcinoma^28^. The variant co-segregates with the disease in multiple affected family members (Fig. 3).

In family 8, the index patient was a 6-year-old girl who had easily controlled diabetes since 2.5 years (Table 1). Her mother had a history of gestational diabetes. We identified a heterozygous missense variant in *HNF4A,* confirming MODY1 (Table 2). To the best of our knowledge, the missense variant p.I463V in *HNF4A* has not been reported previously as a pathogenic variant nor as a benign variant. Polyphen In *silico* analysis predicts a Tolerated/benign effect on protein function when isoleucine is substituted for a valine amino acid at codon 463. However, SIFT analysis predicts a damaging change. HNF4A contains 11 pathogenic missense variants and a low rate of benign missense variation as indicated by a high missense variant Z-score of 1.81 (Table 2, ACMG, PP2). The p.I463V variant has been reported as rare in all populations with -minor allele frequency of 0.00037 or 0.037% except for the Latino population in gnomAD where it is observed in 0.07% of alleles (ACMG, BS1). For these reasons, we classified the variant as VUS (Table 2).

Family 12 was diagnosed with MODY2 (Table 2). The index patient was a 5-year-old Egyptian boy with a maternally inherited missense variant in *GCK* gene c.572G > A (p. R191Q). The child was born to a mother with T2D managed on oral medications. The child’s fasting blood glucose ranged from 7.0–7.7 mmol/L, while his post-prandial blood glucose did not exceed 10.0 mmol/L. Neither mother nor the child required any additional treatment upon diagnosis. The p.R191Q variant occurs at the same amino acid position as the previously classified pathogenic variant p.R191W. GCK contains 122 pathogenic missense variants and three variants within six amino acid positions of the variant p.R191Q, while none have been shown to be benign. The missense variant has been identified in three families from Chile^29^ and Italian children with MODY2^30^.

## Discussion

The genetic basis of MODY in Middle Eastern populations is unknown. In the Gulf Cooperation Council (GCC) region, a study from Oman examined 20 patients with suspected MODY but found no variants in three MODY genes (*HNF4A*, *GCK,* and *HNF1A*) sequenced at Exeter^20^. Similarly, two gene-specific studies conducted in Tunisia identified two variants, one in HNF4A (from 12 patients with diabetes) and the other in the GCK gene (from 23 unrelated patients with diabetes) respectively^17,18^. More recently, a third study from Tunisia utilized targeted NGS and identified four variants from 11 patients suspected to have MODY in *ABCC8*, *HNF1A,* and *GCK*, improving the positive diagnostic rate significantly^19^. There are no studies on MODY published from Kuwait, Bahrain, or Qatar to date. Yet the Middle East and North Africa (MENA) region has the second highest prevalence of diabetes world-wide; and furthermore 14.8% of the adult Kuwaiti population are estimated to live with diabetes^31^. Kuwait also has one of the highest incidence rates of T1D in children^32,33^. This challenge is further confounded in countries like Kuwait and other Gulf Cooperation Council (GCC) by familial clustering of T2D and high rates of consanguineous marriages. It is unclear why academically and clinically lucrative studies on MODY have not been pursued in the Middle East. However, we can postulate three possible reasons for the lack of information on MODY and its genetics in the Middle East; The first may be due to the fact that the diagnosis of MODY is often challenging due to its shared clinical features with T1D and T2D^5^. The second reason may pertain to the technically demanding infrastructure required for the diagnosis of MODY, which is just being addressed in this area of the world. The third reason may be linked to the high propensity of familial clustering in T1D and T2D. For example, a panoply of locally conducted studies has established strong links between positive family history and T2D, with 71% of Arab patients with diabetes in Qatar and 80% of Omani patients having first degree relatives with diabetes^34^; compared to a lower frequency of 33% among Europeans^35^. Lastly, with the extremely high prevalence of obesity in the Middle East and the GCC, specifically in children and young adults^36,37^, the differentiation between MODY and T2D becomes even more clinically challenging. Since ISPAD guidelines suggest that the diagnosis of MODY should be suspected in cases that lack phenotype characteristics of type 2 diabetes, including obesity, the increasing prevalence of obesity may lead to fewer people with MODY being investigated using genetic tests^9^.

In this first-of-its-kind study from the region, we sequenced whole exomes of index patients and first-degree family members from 31 families with suspected MODY based on phenotype and clinical presentation (including negative T1D autoantibodies). We detected 7 MODY gene variants accounting for a positive detection rate of 22.6%. As our genetic testing was not done systematically in the population but focused on highly selected patients with clinically suspected MODY, we cannot establish a reliable estimate of the prevalence of MODY in the Kuwaiti population. In addition, we expect that the prevalence will vary between different ethnic groups, and more population or registry-based studies across the region are needed.

Recent data from a Norwegian nationwide population-based registry^14^ suggests that the prevalence of MODY in children with antibody-negative diabetes may reach 6.5%. Importantly, one-third of these cases with MODY had not been recognized by clinicians. Nationwide screening programs in Europe show similar prevalence for MODY: Poland^38^ has reported 7% MODY cases with a *GCK/HNF1A* ratio of 21, while Germany and Austria^39^ have reported a diagnosis rate of 97% with *GCK/HNF1A* ratio of 2. The United Kingdom^40^ has a MODY diagnosis rate of 27% with *GCK/HNF1A* ratio of 0.61, which closely resembles our data (positive detection rate of 21.8% and *GCK/HNF1A* ratio of 0.5).

*GCK*-MODY2 is the most common subtype of MODY identified in pediatric diabetes clinics^41^. Since it is rarely associated with microvascular or macrovascular complications^42^, affected individuals do not require diabetes treatment or regular follow-up visits ^9,43^. Similarly, MODY 3 cases resulting from variants in *HNF1A* are of significant clinical relevance as they provide a basis for individualized “precision” treatment and management of complications associated with glycemic control. The ISPAD guidelines^9^ recommend that *HNF1α*-MODY be the first diagnostic possibility considered in patients with symptomatic autosomal dominant diabetes. Variants in *HNF1α* show high penetrance^44^ with 79% of carriers developing diabetes before the age of 35. Consistent with this, the two cases diagnosed with MODY3 in our study presented diabetes before the age of 32 years. Exposure to maternal diabetes in utero is considered to accelerate the development of diabetes in the offspring by about 12 years^45^. One of the two MODY3 cases diagnosed in our study shows maternal inheritance of the disease before the age of 14 years. In both families with MODY3 the mothers of the index patients had diabetes. Our analysis also detected two cases of MODY5 driven by the presence of *HNF1β* variants, who had evidence of renal dysfunction (renal cysts and dysplasia requiring renal transplantation in one case and increased levels of serum creatinine in the other). Though diabetes secondary to HNF1β variant develops typically during adolescence or early adulthood^46,47^; two of the MODY5 patients in our study developed diabetes at the age of 4 and 9 years, respectively. The accelerated onset of diabetes could be due to modifiable risk factors such as post-transplantation medications or obesity.

A major challenge in interpreting genetic test results, not limited to MODY, is defining variant pathogenicity. Although we strictly followed the ACMG-AMP variant classification criteria^48^, our study as well as other data sets remain deficient in functional data. Also, there is currently one expert group in ClinGen that has recently started the process of ACMG-based MODY variant classification. Therefore, given the current lack of comprehensive MODY-variant classification expertise, our ACMG-based classification should be interpreted cautiously and may be subject to change in the future.

Despite our success in diagnosing different forms of MODY and thus enabling personalized treatment for patients locally, the refusal of family members to be screened remains a challenge. Another major caveat in this study is that it was based on referrals from physicians rather than utilizing a systematic, nationwide screening of suspected cases based on the ongoing “Childhood Diabetes Registry” in Kuwait. Therefore, our study does not claim to report the prevalence of MODY in the Kuwaiti population.

Moreover, our study shows that, even in highly selected families with suspected MODY that were autoantibody negative, 77.3% did not harbor known MODY-causative genes, although the samples were analyzed using state-of-the-art methodologies of NGS and MLPA at two reputable and independent centers. This suggests that either additional new genes play a role in the MODY-like families presented here or that they are T2D that mimics MODY in terms of familial clustering. Further studies based on broader population screening and deeper whole genome sequencing are being planned to address this vital niche and to unravel the influence of intronic or gene regulatory mutations in the pathogenesis of MODY, if any.

To circumvent these challenges and to contribute more to our understanding of MODY, we established a highly specialized MODY clinic that is appropriately tailored to educate and serve clinicians from Kuwait and the region centrally. Implementing a nationwide national diabetes registry in Kuwait will allow us to address MODY at the population level within the near future with active screening protocols, including more in-depth information on the phenotype of patients and their pedigrees. Increased awareness and improved access to next generation genetic testing will provide interesting new findings to improve our understanding of the genetic makeup of MODY.

In summary, our study provides the first data set on the genotype and phenotype of patients with suspected MODY in Kuwait, based on referrals to a specialized MODY clinic. We describe seven cases representing five MODY gene variants, including three novel variants. Our results demonstrate the importance of addressing MODY by genetic testing to enable precision diabetes management for the affected patients. We plan to integrate MODY screening into the future National Diabetes Registry to capture comprehensive data on MODY in Kuwait, including genotype, phenotype, and long-term outcome in affected patients. The social and ethical implications associated with genetic testing within families and the broader community must be given careful consideration before the implementation of a nationwide project.

## Supplementary Information

Supplementary Figure S1.

## Data Availability

The data set generated during and/or analyzed during the current study are available on reasonable request from the corresponding author.
