# Supplementary material for: Identification of Maturity-Onset-Diabetes of the Young (MODY) mutations in a country where diabetes is endemic
Source: Sci Rep. 2021 Aug 9;11:16060. doi: 10.1038/s41598-021-95552-z (PMC8352960; doi:10.1038/s41598-021-95552-z)
Supplement: Supplementary file 1 — Supplementary Figure S1. [file 41598_2021_95552_MOESM1_ESM.pdf]

Identification of Maturity-Onset-Diabetes of the Young (MODY) mutations in a country where diabetes is endemic

Hessa Al-Kandari[1,2], Dalia Al-Abdulrazzaq[1,3], Lena Davidsson[1], Rasheeba Nizam[4], Sindhu Jacob[4], Motasem Melhem[4], Sumi Elsa John[4], Fahd Al-Mulla[4].

[1]Department of Population Health, Dasman Diabetes Institute, Kuwait
[2]Department of Pediatrics, Farwaniya Hospital, Ministry of Health, Kuwait
[3]Department of Pediatrics, Faculty of Medicine, Kuwait University, Kuwait.
[4]Department of Genetics and Bioinformatics, Dasman Diabetes Institute, Kuwait.

***Correspondence**: Professor Fahd Al-Mulla, Dasman Diabetes Institute, P.O. Box 1180, Dasman 15462, Kuwait, Phone: +965 2224 2999 Ext. 2211, Fax: +965 2249 2436, Email: fahd.almulla@dasmaninstitute.org

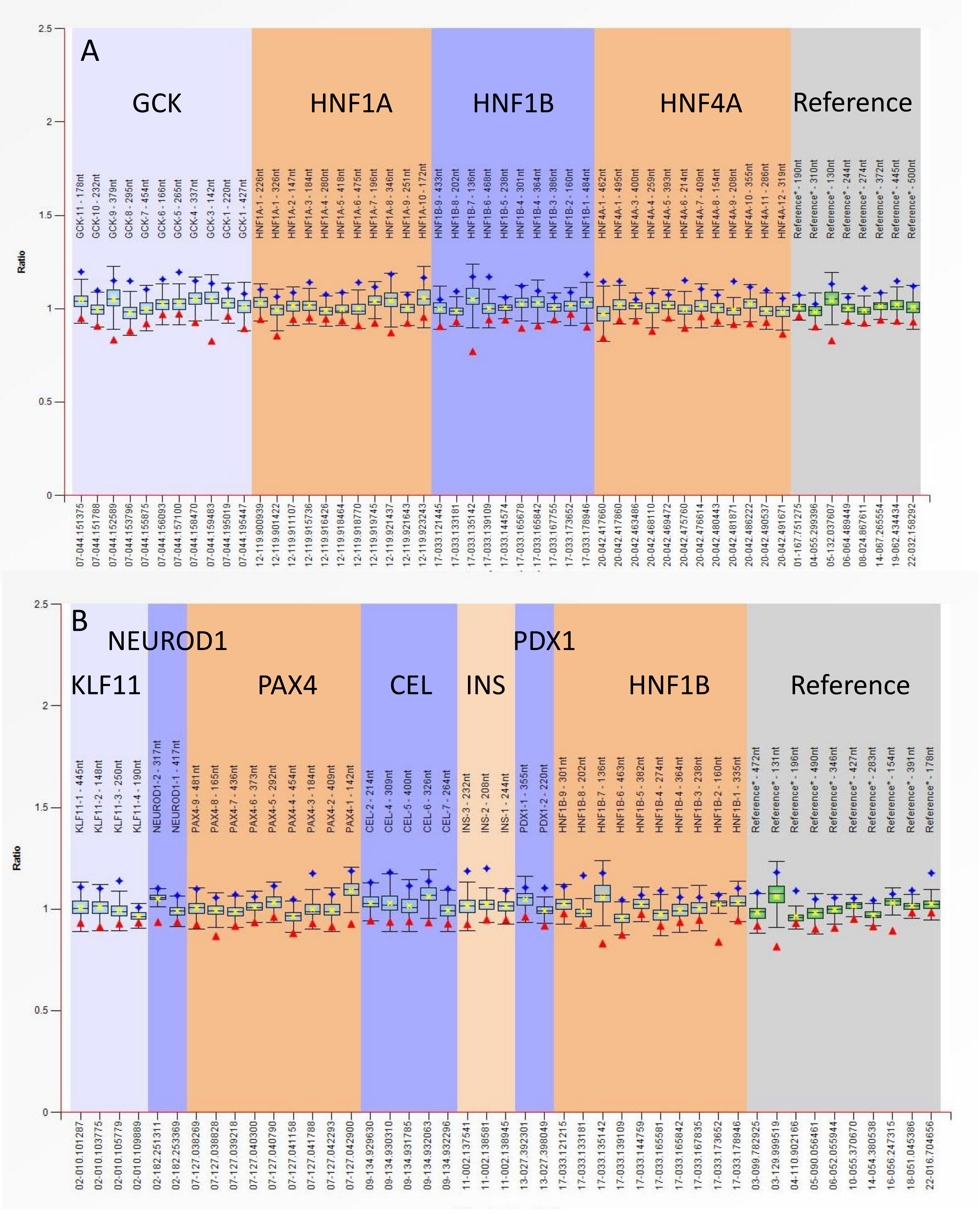

Figure S1: Depicts 95% confidence intervals of all tested cases over the reference samples for (A) MLPA P241-E1 MODY Mix-1 probe, and (B) of MLPA P357-A3 MODY Mix-2 probe as a coloured bar in the ratio chart. The X axis represents gene locations based on hg18 / mapview build 36 and the Y axis shows the Intra and inter-normalized probe ratio
